# A Matter of National Security: The Limitations of Contemporary Bioethical and Public Health Approaches for Addressing Enhanced Warfighters

**DOI:** 10.7759/cureus.94419

**Published:** 2025-10-12

**Authors:** Bilal Irfan, Roberto Sirvent

**Affiliations:** 1 Center for Surgery and Public Health, Brigham and Women's Hospital, Boston, USA; 2 Michigan Alzheimer's Disease Center, University of Michigan, Ann Arbor, USA; 3 Center for Bioethics, Harvard Medical School, Boston, USA

**Keywords:** bioethics, enhanced warfighters, global health concerns, supersoldiers, technology

## Abstract

This piece argues that debates over enhancing warfighters must be reframed from a narrow focus on individual risk, consent, and long-term care to a public health analysis of how national security imperatives allocate risk and foreclose redress. In the United States, legal and institutional deference to the military, exemplified by the bar on service-related tort claims and by authorities permitting presidential waiver of informed consent for investigational products, creates an environment in which even well-designed consent regimes are fragile and options for remedies remain uncertain. Neuroenhancement, framed through a lens of self-strengthening, can also function as a form of discipline, narrowing the space for refusal, doubt, and independent moral judgment that consent procedures presuppose. Bioethics as a field likewise risks drifting toward the psychological burdens of combatants while sidelining the irreparable harms borne by civilians as the health consequences extend beyond soldiers. Civilian populations subjected to heightened military force often absorb the greatest morbidity through destruction of health infrastructure and trauma patterns characteristic of contemporary armed conflict, harms that are still at the margins of the bioethical calculus. Upstream, the supply chain for enhancement risks renewed exploitation of constrained groups, while expanding university-defense partnerships channel research agendas toward operational utility. The piece proposes a health-centered bioethics that measures ethical success by reduced population morbidity, mandates civilian-injury surveillance and protection of medical facilities, and scrutinizes defense funding in academia. Given the limits of international enforcement, bioethics should prioritize building norms and data infrastructures that keep clinics open and populations safer, insisting that health be a first constraint rather than an after-action concern.

## Editorial

Calls to regulate enhanced warfighters may often begin from an ostensibly clinical question: can soldiers safely receive pharmacologic, genetic, or neuro-technological interventions that augment cognition, vigilance, strength, or stress tolerance, and on what terms should consent and long-term care be arranged? Those are important questions, but they understate the regime within which such questions are answered. In the United States, health-related ethics for military personnel are nested inside a legal and cultural apparatus that often subordinates ordinary bioethical commitments to national security. That subordination has concrete medical consequences. It shapes how consent is construed, how adverse effects are documented and compensated, which bodies are exposed to experimentation in the pipeline toward what may be deemed by some as a safe enhancement, and whose health counts when augmented force is projected abroad. An argument that centers health in the debate over super-soldiers may benefit, therefore, from widening its clinical lens: the central medical problem is not individual risk-benefit calculation alone, but the way national security imperatives allocate risk, justify human rights abuses, and foreclose redress across populations.

Bioethics scholarship has begun to parse enhancement in the military context, including careful distinctions among general, informed, and intervention-specific consent, as well as proposals for tracking life-course harms and providing lifetime care for injured service members. Recent perspectives have made clear the pressing need to devote attention to formulating clearer consent architectures and post-deployment obligations in anticipation of the diffusion into practice. That literature is valuable as far as it goes, but it may not always engage the constitutional and institutional settings that decide whether consent claims matter when they conflict with operational priorities. In the contemporary system of the United States, courts and legislatures repeatedly entrench deference to military judgment, and those structures are not ethically inert for health.

The most durable example is a line of Supreme Court and lower-court decisions beginning with *Feres v. United States* (1950), which generally bars military personnel from suing the government for injuries incident to service, including health harms arising from military medical care, research, or operational exposures [1]. Courts have repeatedly invoked this decision to defeat claims by veterans exposed to radiation during nuclear weapons activities and other hazardous programs. While different government programs have provided alternative benefits and presumptive schemes in some contexts, courts emphasize that such administrative compensation does not displace the core bar on raising such suits. The doctrine’s persistence alludes to the idea that even if sophisticated consent frameworks for enhancement were enforced, it would likely be within an environment where tort redress is structurally curtailed. In *Stanley v. CIA* in 1981, for example, the court applied the *Feres* decision to a soldier who was surreptitiously given lysergic acid diethylamide during military programs, concluding that alleged post-discharge failures to warn and monitor did not escape the doctrine, illustrating how military status can eclipse ordinary bioethical protections in litigation [1].

Statute and executive policy further codify exceptions to consent in military health contexts. The President of the United States may waive the informed-consent requirement for administering an investigational medical product to service members in a particular military operation when obtaining consent is not feasible, is deemed to be contrary to the member’s best interests, or is not in the interests of national security [2]. The framework exposes a twofold vulnerability: consent can be waived ex ante under specified conditions, and the order itself provides no avenue for ex post redress if harms occur. From a health-systems perspective, that combination alters risk accounting at the bedside and at the bench, because it reallocates the burden of uncertainty to the soldier’s body without concomitant avenues for adjudicating injuries or ensuring lifetime care outside agency discretion.

Another blind spot in prevailing ethics-related conversations about enhancement is the near absence of those who never consent and yet bear the greatest medical harms: civilian communities subjected to indiscriminate military force. To center health is to track the downstream injury patterns created by enhanced operational tempo or new targeting capabilities. Despite continued airstrikes on medical facilities and hospitals across the world that generate international legal analysis and, at times, internal military investigations, a durable mechanism does not exist for preventing recurrences or compensating public-health losses. As weapons become more discriminating on paper and more sophisticated in practice, bioethics that focuses on soldiers’ consent while ignoring communities’ health will distract from where the bulk of morbidity lies. Destruction of pharmaceutical plants, hospitals, and medical clinics, and the killing and abduction of healthcare workers, have been widely documented and litigated. Even as intelligence rationales shifted in subsequent accounts, the practical result has been the elimination of a major source of healthcare delivery in such resource-constrained settings, with predictable population-level health effects. Such cases demonstrate how national security decisions can precipitate large-scale public health harms that are difficult to count and nearly impossible to remedy through adjudication. If bioethics is to be a health discipline and not only a professional-conduct code, its analysis of super soldiers must reckon with this ledger of harm that rarely enters the consent conversation.

A more candid accounting also requires attention to the supply chain underlying the enhancement of soldiers. Military-ready interventions rarely arrive without preclinical and human testing, and the United States has a long record of exploiting constrained populations to accelerate that path. Prisoners in particular were used extensively in twentieth-century research programs. Awareness and public outrage from such practices catalyzed some regulatory reforms and recommendations for additional protections. Yet the structural temptations remain. When defense priorities heighten demand for novel technologies, the populations with the least leverage over consent are again at risk of being enrolled first. The health stakes are not speculative. Layering exposure to unproven enhancement technologies on top of carceral deprivation could multiply the structural coercion and foreseeable harm in a group with limited access to independent medical advocacy.

The pipeline from laboratory to battlespace also runs through universities, whose collaborations with the Department of Defense may expand as the neuroscience and bioengineering needed for enhancement mature [3]. Such work can be clinically transformative, but it also pulls academic inquiry into a funding ecosystem where the metrics of success include operational utility. Bioethics must ask not only whether institutional review boards are robust, but also how corporate and defense dollars shape topic selection, disincentivize critical scholarship on the harms of militarized research, and normalize the framing of enhancement as an inevitable horizon to be managed rather than a policy choice to be contested. Meanwhile, the arms industry’s revenues continue to rise amid global conflict, a reminder that the political economy surrounding enhancement has strong incentives to bring tools to market, doctrine, and theater quickly.

If debate is to remain anchored in health, the medical sequelae of specific enhancement modalities deserve close attention, as more invasive modalities may raise distinct hazards. Advocates may sometimes reply that these are precisely the reasons to build stronger consent and surveillance systems for enhanced warfighters. Yet in practice, the same legal and cultural currents that protect the chain of command from judicial scrutiny could also weaken those systems. Human subjects regulations, at times, may contemplate non-consensual administration of investigational products in narrow circumstances tied to national security, and at scale, the rights of refusal may quickly yield to force-protection. There have already been worries about the truncation of the threat of tort enforcement for substandard care or undisclosed risks. In that ecosystem, even robust internal ethics codes for military clinicians will be tested at their breaking point when clinical judgment collides with operational orders.

There is also a subtler health transformation that enhancement invites: by expanding the physiological envelope of human operators, it shifts strategic doctrine toward longer, more aggressive, or more remote operations where commanders assume less attrition. The availability of wakefulness agents and fatigue countermeasures may lead to longer military missions. Similar dynamics could follow with cognitive-bias mitigation drugs, stress-blunting neuromodulation, or exoskeletons that reduce immediate musculoskeletal load. On the battlefield, that may decrease acute injury for soldiers while increasing the intensity and duration of engagements that generate civilian trauma patterns known from other conflicts: multi-site blast trauma, complex burns, penetrating head and torso injuries, and cascading destruction and devastation of local health systems when facilities are destroyed or embargoed [4]. A bioethics that limits itself to the soldier’s health and privileges moral appeals to national security may forget such downstream and direct consequences.

All of this also raises the question of the audience. Contemporary understandings of the Western-dominated bioethics, as a field, point to it emerging in part to protect patients from powerful institutions, and it tells origin stories about post-war accountability. In the defense sector, however, bioethics is more often advisory than countervailing. Industry-funded conflicts of interest routinely distort research agendas in medicine, and bioethics as a field has weaker, less uniform conflict-of-interest policies than the biomedical journals whose practices it critiques. The same actors that profit from war also fund research centers, endow hospital chairs, and sponsor conferences where enhancement is framed as a technical inevitability [5]. Those entanglements matter for health. They can starve critical scholarship, crowd out epidemiology on civilian harm, and naturalize the idea that the primary ethical constituency in enhancement is the soldier’s autonomy rather than the communities whose clinics and bodies are transformed by augmented violence.

One further concern is distributive: whose suffering becomes legible to bioethics when violence is organized through policy. Too often, the field’s gaze settles on the psychological burdens borne by combatants who perpetrate harm while leaving the medical realities of those harmed at the periphery. Work that foregrounds the distress of perpetrators can function as a kind of rhetorical violence when it recenters the moral narrative around the well-being of agents of harm and dilutes attention to the people whose clinics, hospitals, and cities were destroyed, whose kin were killed, and whose futures were medically foreclosed. There may be limited contexts where prioritizing the immediate examination of the mental health of perpetrators over other justice-oriented solutions is warranted, perhaps for prevention, accountability, or institutional reform, but such inquiry requires careful ethical framing to avoid transforming perpetrators into primary victims in the story bioethics tells. A health-centered approach could adopt an ethics of attention proportional to vulnerability and irreversibility of harm, ensuring that the baseline unit of moral analysis is the civilian body that never consented, not the armed body that did.

What then should a global health-centered bioethics approach argue? First, that any credible analysis of enhanced warfighters must adopt a public-health frame alongside an individual-rights frame. The metric of ethical success is not merely whether a consent form is signed under military hierarchy, but whether aggregate morbidity and mortality are reduced for everyone affected by such enhanced force. That standard demands routine inclusion of civilian-injury surveillance and the protection of medical facilities as outcome measures in the ethics of enhancement. The broader documentation of attacks on health care in conflict zones shows that such measures are not abstractions. When those facilities are erased, the health debt runs for years. Second, bioethics should be forthright about redress. So long as the current sociopolitical climate persists, the likely path for harmed soldiers will be administrative remedies, not tort. Any rollout of enhancement technologies should therefore be paired, from inception, with presumptive service-connection rules, funded specialty clinics, and transparent adverse-event registries administered outside the chain of command. That is not a substitute for judicial accountability, but it at least acknowledges the legal terrain on which clinicians and patients actually stand. Third, academic medicine should revisit the terms of its engagement with defense funding in enhancement-relevant domains. Where programs primarily serve military ends without clear civilian health benefits, universities should be prepared to decline sponsorship or, at a minimum, require genuinely independent ethical review bodies with public reporting mandates and wall off clinical data from operational military use that risks violating human rights. The public-health rationale is simple: the same pipelines that speed enhancement to the field can bypass the kinds of post-market surveillance and registries that protect patients in civilian medicine. A clinical culture that tolerates that asymmetry because the patient is a soldier accepts, in effect, a two-tiered ethics.

Finally, bioethics should acknowledge its limits. International humanitarian law has not reliably constrained great-power conduct when national security claims are invoked, and the United Nations structure makes symmetric enforcement unlikely. In that environment, narrowly technical debates about the moral status of the enhanced body may satisfy professional curiosity while leaving untouched the health realities of populations living under drones, missiles, military occupations, and sieges. The more urgent bioethical work is to help build norms and data infrastructures that keep clinics open, track civilian casualties with clinical rigor, collaborate with communities mobilizing against imperialism, and defend the independence of humanitarian health providers. That work will often be at cross-purposes with enhancement regimes that promise greater lethality and casualties. History suggests that when national security is the highest law, bioethics will be invited to consult after the fact. The task now is to insist that health be the first constraint, not the after-action report.
